# Bilirubin augments Ca^2+^ load of developing bushy neurons by targeting specific subtype of voltage-gated calcium channels

**DOI:** 10.1038/s41598-017-00275-9

**Published:** 2017-03-27

**Authors:** Min Liang, Xin-Lu Yin, Hai-Bo Shi, Chun-Yan Li, Xin-Yi Li, Ning-Ying Song, Hao-Song Shi, Yi Zhao, Lu-Yang Wang, Shan-Kai Yin

**Affiliations:** 1 Department of Otorhinolaryngology, Shanghai Jiao Tong University Affiliated Sixth People’s Hospital, 600 Yishan Road, Shanghai, 200233 P. R. China; 2 Programs in Neurosciences & Mental Health, SickKids Research Institute and Department of Physiology, University of Toronto, Toronto, ON M5G 1X8 Canada; 3 Department of Otorhinolaryngology, West China Hospital, Sichuan University, No. 37, GuoXueXiang, Chengdu 610041 P. R. China

## Abstract

Neonatal brain is particularly vulnerable to pathological levels of bilirubin which elevates and overloads intracellular Ca^2+^, leading to neurotoxicity. However, how voltage-gated calcium channels (VGCCs) are functionally involved in excess calcium influx remains unknown. By performing voltage-clamp recordings from bushy cells in the ventral cochlear nucleus (VCN) in postnatal rat pups (P4-17), we found the total calcium current density was more than doubled over P4-17, but the relative weight of VGCC subtypes changed dramatically, being relatively equal among T, L, N, P/Q and R-type at P4-6 to predominantly L, N, R over T and P/Q at P15-17. Surprisingly, acute administration of bilirubin augmented the VGCC currents specifically mediated by high voltage-activated (HVA) P/Q-type calcium currents. This augment was attenuated by intracellular loading of Ca^2+^ buffer EGTA or calmodulin inhibitory peptide. Our findings indicate that acute exposure to bilirubin increases VGCC currents, primarily by targeting P/Q-type calcium channels via Ca^2+^ and calmodulin dependent mechanisms to overwhelm neurons with excessive Ca^2+^. Since P/Q-subtype calcium channels are more prominent in neonatal neurons (e.g. P4-6) than later stages, we suggest this subtype-specific enhancement of P/Q-type Ca^2+^ currents likely contributes to the early neuronal vulnerability to hyperbilirubinemia in auditory and other brain regions.

## Introduction

Neonatal unconjugated hyperbilirubinemia and clinical jaundice occur in up to 60% of full-term newborns and almost all preterm infants^1–3^. When untreated, acute bilirubin encephalopathy may progress to kernicterus, characterized by auditory impairment (including deafness), movement disorder (dystonia and/or athetosis), ocular movement impairment and dental enamel dysplasia of the deciduous teeth^4, 5^. Auditory neurons are highly vulnerable to bilirubin-induced neurotoxicity due to excessive glutamate release and hyperexcitation^6–8^. The main effect of bilirubin is associated with intracellular Ca^2+^ overload and activation of apoptotic pathways and necrosis^4, 9, 10^. However, the routes underpinning calcium overload by bilirubin remain elusive.

Numerous studies have implicated Ca^2+^ as a mediator of neurotoxicity. A major route for Ca^2+^ overload is excess Ca^2+^ inflow into cells through voltage-gated calcium channels (VGCCs). VGCCs are usually activated during action potentials (APs) and subthreshold depolarizing signals^11^ to permeate Ca^2+^, which acts as a ubiquitous second messenger to initiate signaling cascade to regulate gene transcription and translation as well synaptic transmission and plasticity. These channels can be classified into T-, L-, N-, P/Q- and R-types^12–16^. T-type channels are low voltage-activated (LVA) channels transiently activating at hyperpolarized membrane potentials and sensitive to non-dihydropyridine compound mibefradil^17–19^. High voltage-activated (HVA) channels are activated at more depolarized potentials and classified into: L-type channels that are blocked by dihydropyridine antagonists; N- and P/Q-type channels that are sensitive to ω-Conotoxin GVIA (ω-CTx GVIA) and ω-Agatoxin IVA (ω-Aga IVA), respectively^20–24^. Low concentration of NiCl_2_ inhibits some native R-type channels^17, 25, 26^.

Both LVA and HVA VGCCs are present in bushy neurons of the anterior ventral cochlear nucleus (AVCN)^27^;, which are among the most vulnerable neurons to bilirubin neurotoxicity^9^. Bushy neurons have large round soma of small dendritic fields and serve as the first central station to project excitatory inputs to other major auditory nuclei critical for sound localization^28–33^. The morphological characteristics and important roles of bushy neurons make them an ideal model to study distribution of calcium channels during development and bilirubin-induced effects. In this study, we showed that the relative weight of each subtype to the total VGCC current over the early development diverged from being homogeneous to heterogeneous, and surprisingly, bilirubin was found to preferentially increase P/Q channel currents without affecting other VGCCs. These novel results implicate P/Q-type calcium channel as the primary VGCC subtype in bilirubin-induced Ca^2+^ overload and “hyperexcitation”.

## Results

### VGCC Currents in Bushy neurons

**Figure 1:**
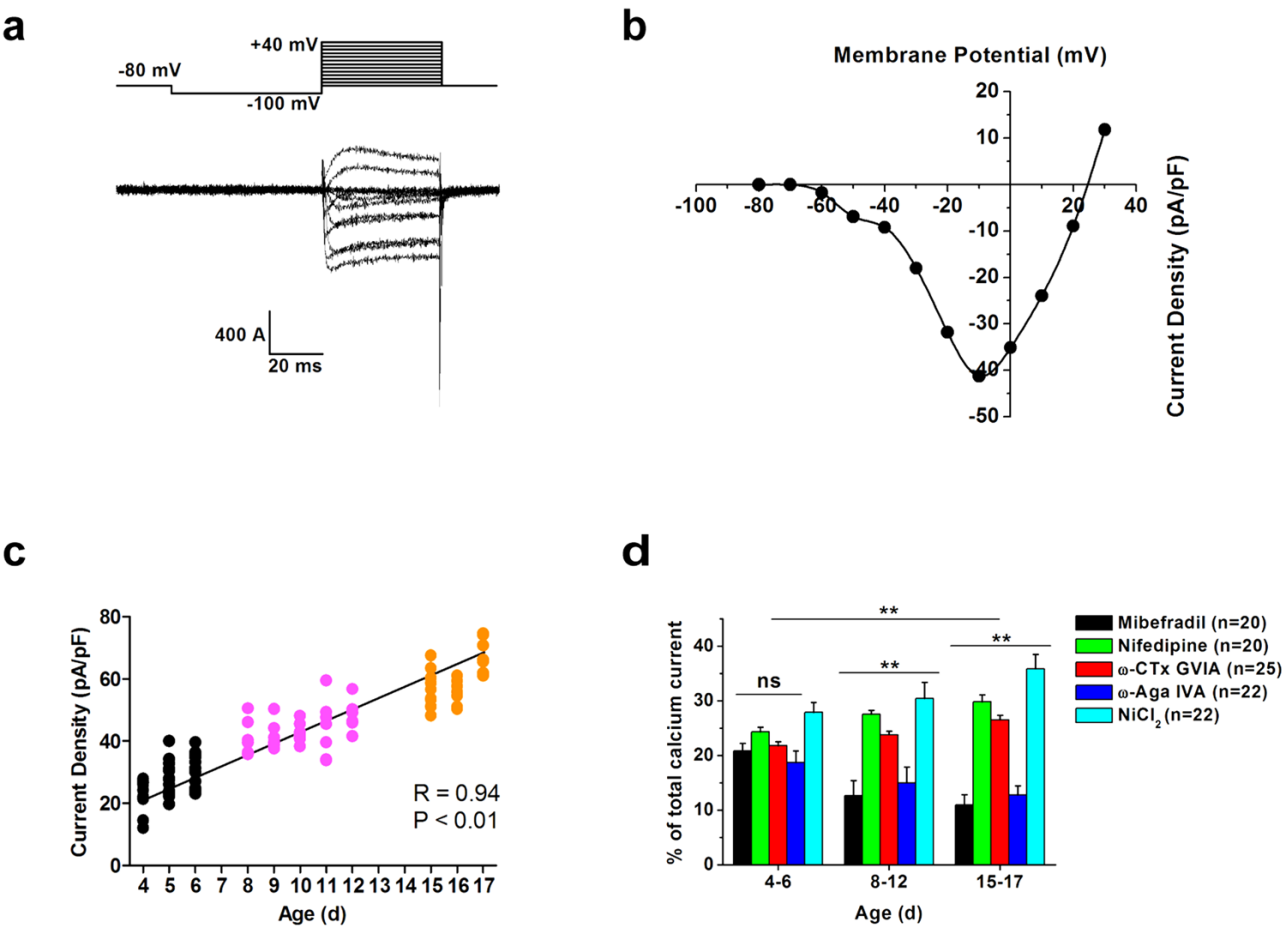
VGCC currents in Bushy neurons. (**a**) Whole-cell currents recorded from a bushy neuron in AVCN. The cell was held at −80 mV and hyperpolarizing to −100 mV before depolarizing to various test potentials from −100 to + 40 mV in 10 mV increment at 5 sec intervals. (**b**) Current-voltage relationships for data in A measured at the peak current amplitudes showed both LVA and HVA VGCC currents. The peak inward current activated positive to about –70 mV and peaked at about –10 mV. Charge carrier, 5 mM Ba^2+^. (**c**) Correlation between the age and the total VGCC current density in bushy neurons. Bushy neurons were divided into three age groups (P4-6, black circles; P8-12, magenta circles; P15-17, orange circles). (**d**) Developmental changes in VGCC current in bushy neurons. The cells were bathed in 5 mM external barium. The extent of inhibition by Mibefradil (1 µM; black bars; n = 20), nifedipine (10 µM; green bars; n = 20), ω-CTx GVIA (1 µM; red bars; n = 25), ω-Aga IVA (500 nM; blue bars; n = 22) and NiCl_2_ (50 µM; cyan bars; n = 22) was quantified and compared to show the contribution of each antagonist-sensitive calcium channels in three age groups (P4-P6, P8-P12 and P15-P17). Five One-way ANOVA tests were further used to determine whether there were any significant differences between the fraction of each subtype current in three age groups. **P < 0.01, ns, not significant, two-way ANOVA with Bonferroni post hoc test.

We first evoked VGCC currents by a series of voltage steps ranging from −100 to + 40 mV (holding potential at −80 mV, prepulse hyperpolarizing at −100 mV for 50 ms) from bushy neurons, as exemplified by the recording using 5 mM Ba^2+^ as the charge carrier (Fig. 1a). The inward current began to appear at −60 mV but showed substantial inactivation, typical of LVA VGCCs. With incremental increase in voltage steps, currents increase in their amplitude but inactivation became less apparent, indicating that HVA currents were activated. Indeed, when we plot the current (I)-voltage (V) relationships, LVA and HVA currents have distinguishable voltage-dependence with the peak at −40 and −10 mV, respectively (Fig. 1b). These inward currents were blocked by application of 200 µM CdCl_2_ (data not shown), confirming that VGCCs mediate these currents carried by Ba^2+^.

To determine whether there was any developmental change in the total current density in bushy neurons, we recorded VGCC currents in three different age groups. We plotted all individual current amplitude values as the function of age of the animals, and found the calcium current density was positively correlated with the age. At P4-6, the mean current density was 27.3 ± 0.9 pA/pF (n = 39, 19 rats). However, the current density increased dramatically to 43.3 ± 1.0 pA/pF (n = 36, 12 rats) at P8-12 and 59.0 ± 1.3 pA/pF (n = 36, 12 rats) at P15-17 (Fig. 1c). We next examined if there were developmental changes among multiple calcium channel types. Subtype-specific blockers were applied using randomized sequence to avoid time-dependent changes in the sensitivity of VGCC currents to blockers, namely mibefradil, dihydropyridine, ω-CTx GVIA, ω-Aga IVA and Ni for T-, L-, N-, P/Q and R subtype, respectively. Overall results demonstrated significant differences in the effects of these blockers among three age groups (Two-way ANOVA, F = 3.62, P = 0.001). Bonferroni post-hoc tests showed that at P4-P6, T-, L-, N-, and P/Q-type of calcium channels were present at cell bodies with each subtype contributing equally to the total current. However, significant difference was found among the weight of each subtype at P8-12 and P15-17. One-way ANOVA Bonferroni post-hoc tests showed that the fraction of T-and P/Q-type current amplitudes showed age-dependent decreases between P4-6 and P15-17 with the former being relatively lower than other types of VGCC current. The fraction of L-type and N-type current tended to increase, becoming predominant contributors to the total current at P15-17 comparing to P4-6. The results demonstrate that the relative weight of each subtype current to the total current over the early development diverges from being homogeneous at P4-6 to heterogeneous at P8-12 and P15-17 with T- and P/Q-type current being significantly less prominent than other subtypes current (Fig. 1d).

### Enhancement of VGCC currents by Bilirubin

**Figure 2:**
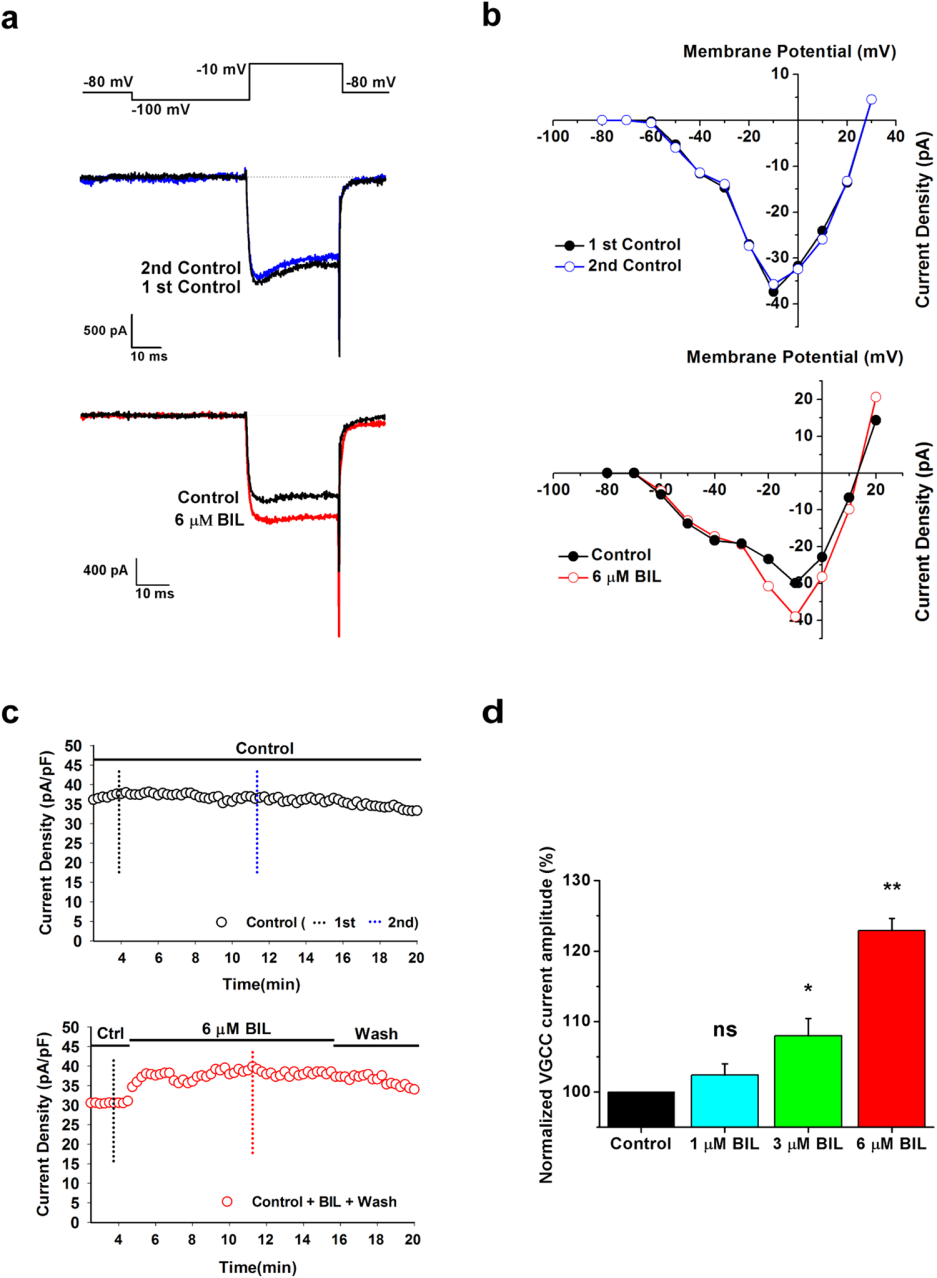
Bilirubin induced increase of VGCC currents. (**a**) Representative traces at −10 mV for currents obtained in the absence (2nd panel, black trace) or presence of 6 μM bilirubin (2nd panel, red trace) in the beginning and after about 11 min of the recording. Bilirubin-induced increase was not due to run-up of VGCC currents because responses in the control solution after 11 min (2nd control) of the recording remained stable (1st panel, blue trace) as illustrate by recording from another cell. Each current was elicited by a 40 ms step to −10 mV. Using Ba^2+^ as charge carrier, the peak inward current apparently increased by bilirubin, but showed no significant inactivation. (**b**) Current–voltage relationships from an experiment before and after application of 6 μM bilirubin. In each case, the holding potential was −80 mV. Note the increase in maximum amplitude with no shift after application of bilirubin. (**c**) Representative time course of the effect of bilirubin. The control solution was applied for 5 min, followed by the bath application of 6 μM bilirubin for 10 min and a washout for another 5 min (red circles). By comparison, control currents were measured in standard solution for 20 min (black circles). Currents were evoked by 40 ms step potentials to −10 mV at 15 s intervals. (**d**) Normalized VGCC current amplitude (%) in the presence of bilirubin at different concentrations: 1 μM (cyan bar, n = 5), 3 μM (green bar, n = 22) and 6 μM (red bar, n = 41). Bilirubin concentrations of 3 μM and 6 μM could induce a significant increase in the amplitudes of calcium currents. For the control group, only a standard solution was applied. *P < 0.05, **P < 0.01, ns, not significant, one-way ANOVA with Bonferroni post hoc test.

To study whether and how bilirubin affects the amplitude of VGCC currents and their voltage dependence, we evoked VGCC currents with the same voltage paradigm as in Fig. 1 to obtain control I-V relationship in rats at P4-12. Subsequently, we used single test pulses from −100 to −10 mV to monitor time-dependent changes in currents following application of 6 μM bilirubin (Fig. 2a–c) and determined the I-V relationship again after bilirubin equilibrated. We found that current amplitudes began to grow in 2 min and usually reached to the maximum within 4-6 min, and this increase was partially reversed after wash (Fig. 2a and c). In contrast, control experiments showed that VGCC currents were stable over the same recording period of time, suggesting that bilirubin-induced increase was not due to run-up of VGCC currents. Comparison of two sets of IV relationships before and after bilirubin application revealed that only the HVA component of VGCC currents was selectively enhanced. We further investigated the effect of bilirubin on VGCC currents at different concentrations (1 µM, 3 µM and 6 µM), and found that the current amplitude after 10-min application of 1 µM bilirubin was not significantly different from that in the control solution. However, the peak current was increased to 108.0 ± 2.4% at 3 μM (P = 0.019, n = 22) and 122.9 ± 1.6% at 6 μM bilirubin (P = 3.26 × 10^−14^, n = 41) (Fig. 2d). We found subtle difference in the relative effect of 6 μM bilirubin over the age range used in these experiments (124.3 ± 2.7% at P4-P6, n = 18; 121.8 ± 1.8% at P8-P12, n = 23; P = 0.445). These results indicate that acute exposure to bilirubin dose-dependently boosts currents mediated by VGCCs in bushy neurons.

### Effects of Bilirubin on voltage-dependence of activation and inactivation of VGCC currents

**Figure 3:**
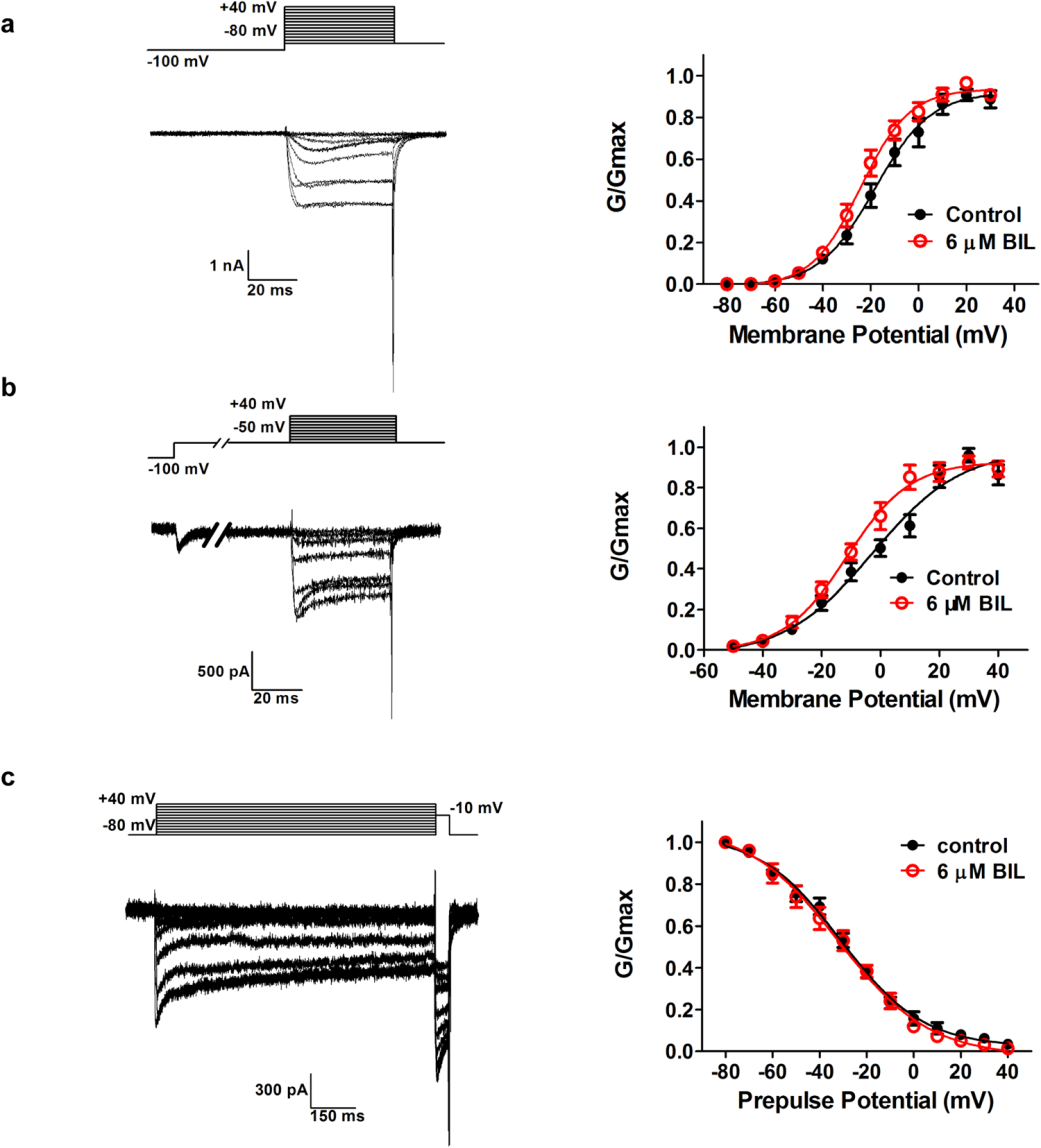
Effects of Bilirubin on activation and steady-state inactivation of VGCCs in bushy neurons. (**a**) Representative traces recorded from a bushy neuron obtained by a 50 ms prepulse at −100 mV to various potentials followed by a 40 ms test pulse to + 40 mV (10 mV increments) are shown (left). Activation of VGCCs in control (black circles) and bilirubin group (red circles) were then fit by a Boltzmann equation. Exposure of bushy neurons to 6 μM bilirubin caused a shift in steady-state activation toward more negative potentials (right). (**b**) Activation of the HVA current was measured on stepping to a test voltage from −50 mV to + 40 mV (10 mV increments) following a prepulse hyperpolarizing at −100 mV for 50 ms and step to −50 mV for 100 ms. The left figure shows representative traces. Activation of VGCCs in control (black circles) and bilirubin group (red circles) were fit by a Boltzmann equation. Exposure of bushy neurons to 6 μM bilirubin caused a left shift on activation curve (right). (**c**) Representative traces recorded from a bushy neuron obtained by a 1 s prepulse to various potentials (10 mV increments) followed by a 50 ms test pulse to −10 mV are shown (left). Steady-state inactivation of VGCCs in control (black circles) and bilirubin group (red circles) were fit to a Boltzmann relationship. Exposure of bushy neurons to 6 μM bilirubin caused no significantly effect on the steady-state inactivation curve (right).

Further experiments and analyses were performed to characterize the effects of the bilirubin on the activation and inactivation properties of VGCCs in rats at P4-12. To generate activation curves, we measured the steady-state currents from data in Fig. 1 and transformed I-V relationship to conductance-voltage curves (G-V), which were fit with the Boltzmann function. We found that the activation curve was slightly shifted toward more hyperpolarization potentials in response to 6 µM bilirubin at a holding potential of −80 mV (Fig. 3a), and the half-activation voltage V_0.5_ of which changed from −18.4 ± 1.7 mV in control to −24.3 ± 1.2 mV in bilirubin (p = 0.002, n = 11) with no change in the slope factor (control, k = 11.6 ± 1.6; bilirubin, k = 10.3 ± 1.1; p = 0.165, n = 11). To differentiate the effect of bilirubin on LVA and HVA calcium currents, we delivered a prepulse depolarizing step to −50 mV to first inactivate LVA VGCCs before giving each test pulses to activate HVAs. Under such conditions, we found that exposure to 6 µM bilirubin again caused a shift in steady-state activation of HVA currents toward more negative potentials (V_0.5_ values: −2.56 ± 2.6 mV in control and −11.33 ± 1.9 mV in bilirubin, p = 0.006, n = 11) (Fig. 3b), indicating that HVA VGCCs are the likely target of modulation by bilirubin.

The steady-state inactivation was examined with a two-pulse protocol, the membrane potential was conditioned to different potentials (varied from −80 to + 40 mV, with + 10 mV increments) for 1 s and then depolarized to a fixed test potential of −10 mV. VGCC currents from bushy neurons in the presence and absence of bilirubin exhibited a similar voltage dependence of inactivation when fit with the Boltzmann function (Fig. 3c). The V_0.5_ values were −31.0 ± 1.9 mV (k = 17.5 ± 1.9) for control solution and −32.0 ± 2.5 mV (k = 19.3 ± 2.5) for bilirubin group (p = 0.534, n = 10). Thus, exposure to bilirubin in these neurons increased the amplitude of VGCC current by enhancing voltage dependent activation without affecting steady-state inactivation of VGCC channels.

### Pharmacological identification of VGCC subtypes sensitive to bilirubin

**Figure 4:**
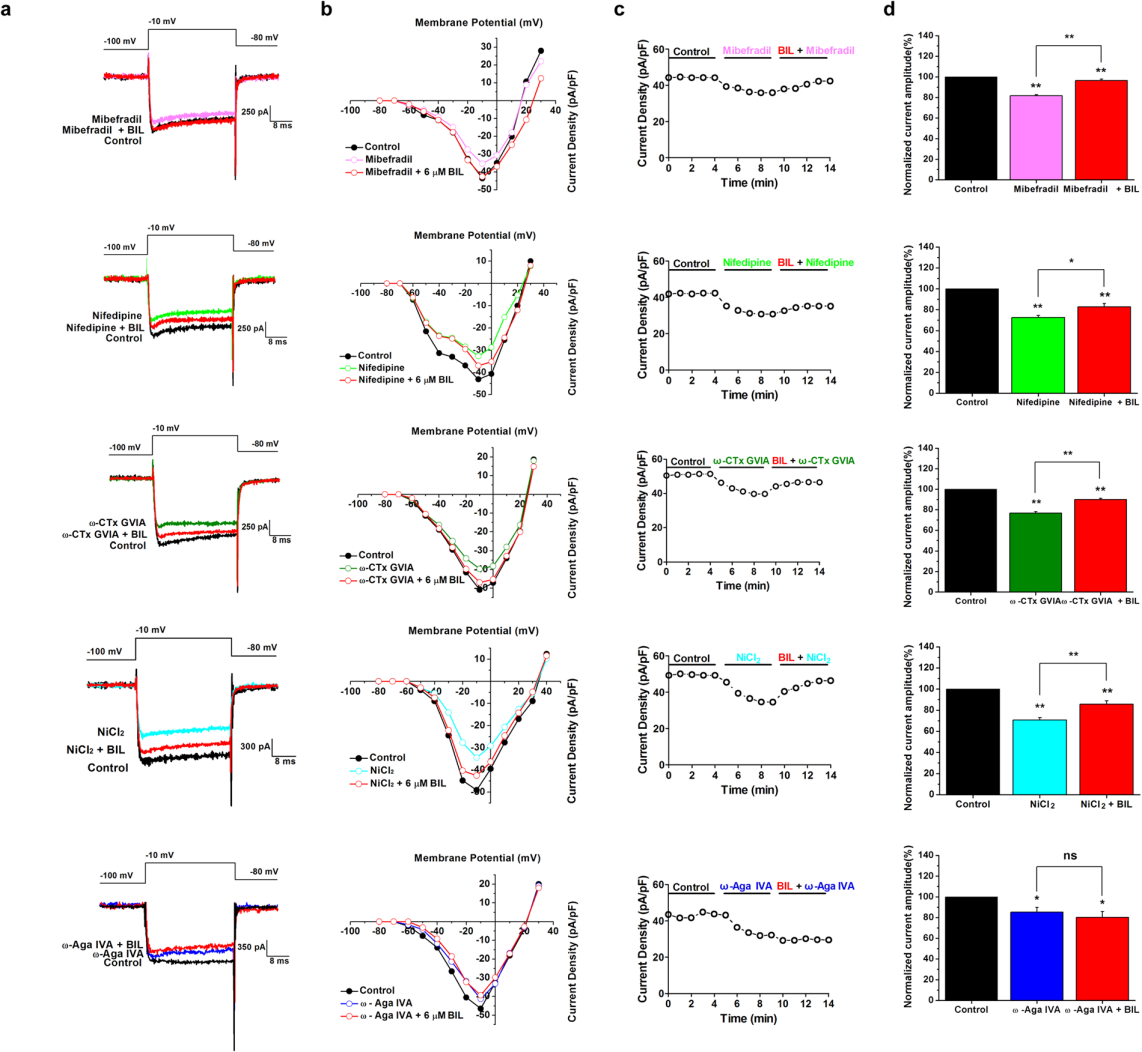
Pharmacological isolation of the VGCC current component enhanced by bilirubin. (**a**) Example traces are shown in control (black traces), during application of 1 µM Mibefradil (top panel, magenta trace, n = 7), 10 µM Nifedipine (2nd panel, green trace, n = 7), 1 µM ω-CTx GVIA (3rd panel, olive trace, n = 7), 50 µM NiCl_2_ (4th panel, cyan trace, n = 7), or 500 nM ω- Agatoxin IVA (bottom panel, blue trace, n = 7), respectively, and subsequent application of 6 µM Bilirubin (red traces), elicited by step depolarizations to −10 mV from a hyperpolarizing potential of −100 mV. (**b**) Current-voltage relationships from data in A measured at the peak current density showed calcium currents in control (black traces), Mibefradil (top panel, magenta trace), Nifedipine (2nd panel, green trace), ω-CTx GVIA (3rd panel, olive trace), NiCl_2_ (4th panel, cyan trace), or ω- Agatoxin IVA (bottom panel, blue trace), respectively, and further addition of 6 µM bilirubin (red traces). (**c**) Time course of the effect of control condition, each antagonist and co-application of bilirubin. The calcium current density is plotted against time. (**d**) The normalized VGCC current amplitude before (black bars), during application of Mibefradil (top panel, magenta bar), Nifedipine (2nd panel, green bar), ω-CTx GVIA (3rd panel, olive bar), NiCl_2_ (4th panel, cyan bar), or ω- Agatoxin IVA (bottom panel, blue bar), respectively, and combined application of 6 μM bilirubin (red bars). Charge carrier: 5 mM Ba^2+^. *P < 0.05, **P < 0.01, ns, not significant, one-way ANOVA with Bonferroni post hoc test.

To investigate which HVA calcium channel subtypes contribute to the enhancement of bilirubin-induced increase in VGCC currents in bushy neurons, we pharmacologically studied different VGCC current components by removing one subtype each time before bilirubin application, that is, to apply the specific P/Q-type calcium channel blocker ω-Aga IVA, N-type channel blocker ω-CTx GVIA, the selective L-type channel blocker Nifedipine, R-type channel blocker NiCl_2_, or the T-type channel blocker Mibefradil at high concentrations in different subset of cells. We evoked VGCC currents with repeated voltage steps and intermittent single voltage steps to monitor the time course of bilirubin effects. Following the establishment of stable antagonists-induced attenuation of VGCC currents (~5 min), we co-applied bilirubin to test its effectiveness in augmenting the total current. The maximum current amplitude was significantly increased by bilirubin to 118.3 ± 2.1% (P = 3.37 × 10^−8^, n = 7, in 1 µM Mibefradil), 114.2 ± 2.2% (P = 0.014, n = 7, in 10 µM Nifedipine), 117.4 ± 2.4% (P = 2.24 × 10^−7^, n = 7, in 1 µM ω-CTx GVIA), or 120.8 ± 2.0% (P = 0.001, n = 7, in 50 µM NiCl_2_), respectively. Surprisingly, we found that only in the presence ω-Aga IVA, bilirubin failed to enhance VGCC currents. The currents were inhibited to 85.7 ± 4.4% (p = 0.046, n = 7) of the control condition after 500 nM ω-Aga IVA application, in line with our previous results. Subsequently, we added 6 μM bilirubin to the perfusion solution and found that bilirubin had no effects on calcium currents with the mean current amplitude being 92.1 ± 4.3% (P = 1, n = 7) at co-application of ω-Aga IVA and bilirubin. Of seven neurons in which calcium current was studied, two cells exhibited <3% increase of calcium current, while other cells had no effect on the maximum current amplitude after bilirubin treatment in the presence of 500 nM ω-Aga IVA. These results indicate that ω-Aga IVA occluded the bilirubin-induced increase of the VGCC currents, demonstrating that the current mediated P/Q-subtype VGCCs is the primary substrate underlying bilirubin-induced enhancement of calcium currents (Fig. 4a–d).

**Figure 5:**
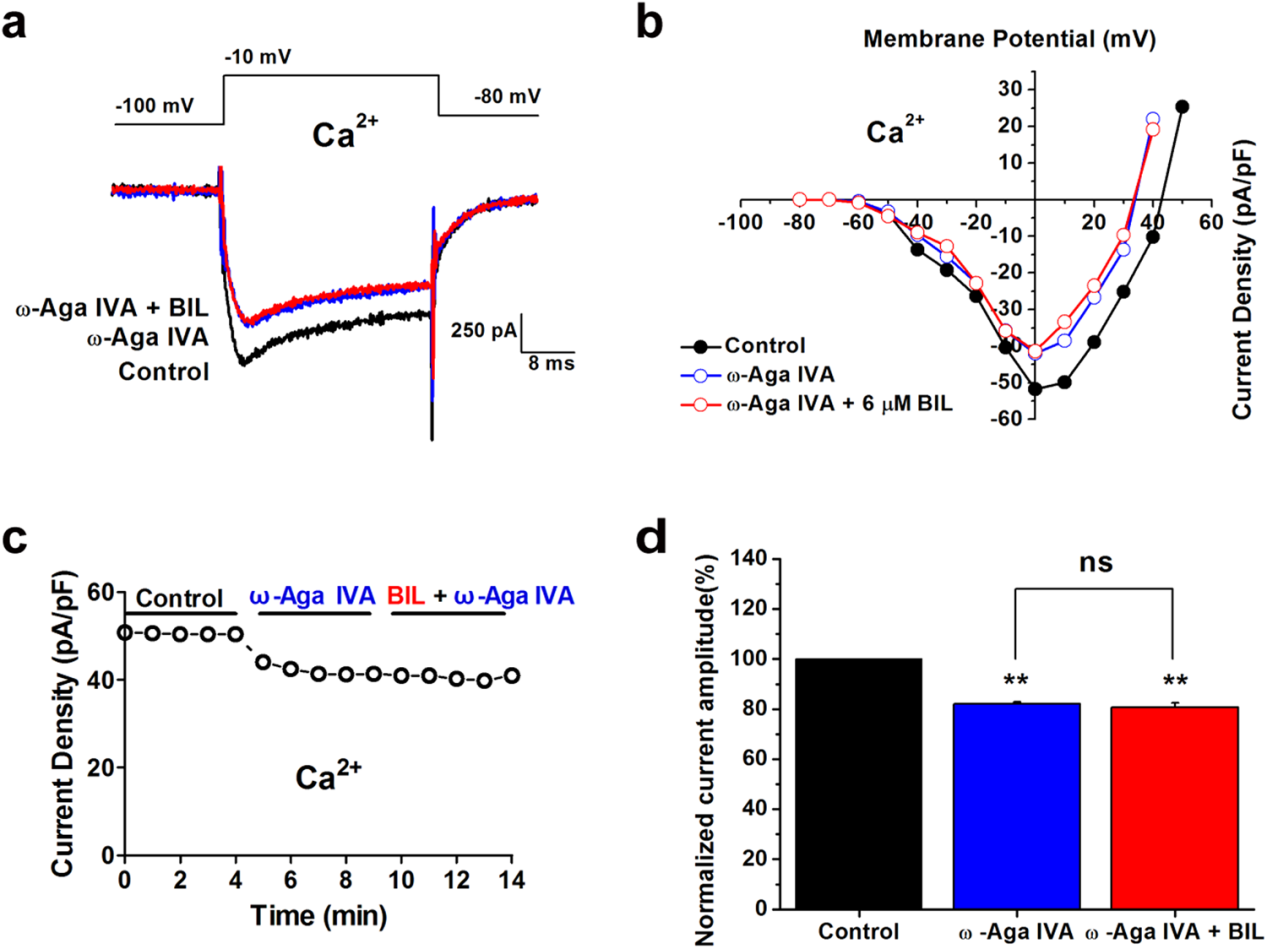
The bilirubin-induced enhancement of VGCC current was primarily mediated by P/Q-type channels using Ca^2+^ as the charge carrier. (**a**) Representative traces in control condition (black trace) and bath application of (blue trace), followed by combined application of 500 nM ω- Agatoxin IVA and 6 μM bilirubin (red trace). Each current was elicited by a 20 ms test pulse to −10 mV from −100 mV. Note a faster rate of inactivation while Ca^2+^ was used as the charge carrier. (**b**) Current-voltage relationships from data in A measured at the peak current density showed HVA calcium currents in control solution (black trace), 500 nM ω- Agatoxin IVA solutions (blue trace), and 500 nM ω- Agatoxin IVA with bilirubin solutions (red trace). The inward currents peaked at about 0 mV. Charge carrier was 2 mM Ca^2+^. Note that switching the type of permeate ions also resulted a shift in the reversal potential to more positive voltages with Ca^2+^ compared with Ba^2+^ as the charge carrier (E_rev_ Ca^2+^ >E_rev_ Ba^2+^). (**c**) Time course of the effect of P/Q-type calcium channel on bilirubin-induced hyperexcitation. Calcium current density measured from recordings in the same neuron from data A and B is plotted against time before, during application of 500 nM ω- Agatoxin IVA, and combined application of 500 nM ω- Agatoxin IVA and 6 μM bilirubin. Data were collected using 2 mM Ca^2+^ as charge carrier. (**d**) The normalized VGCC current amplitude before (black bar), during application of 500 nM ω- Agatoxin IVA (blue bar), and combined application of 500 nM ω- Agatoxin IVA and 6 μM bilirubin (red bar) from six neurons. Charge carrier, 2 mM Ca^2+^. **p < 0.01, ns, not significant, one-way ANOVA with Bonferroni post hoc test.

To reconsolidate this result, we substituted 2 mM Ca^2+^ for 5 mM Ba^2+^ as the carrier ion (Fig. 5a–d) and found that 500 nM ω-Aga IVA decreased current density to 82 ± 0.8% (p = 1.12 × 10^−7^, n = 6) but not reversed upon co-application of bilirubin, which was 98.5 ± 1.4% of only ω-Aga IVA application (p = 1, n = 6, Fig. 5a–d). Based on these observations, we suggest that, P/Q-type calcium channel, albeit of their relatively small contribution to the total current, likely plays a dominant role over other HVA channels in contributing to bilirubin-dependent enhancement of Ca^2+^ currents in developing auditory neurons.

### Ca^2+^ buffer EGTA prevents bilirubin-induced enhancement of calcium currents

**Figure 6:**
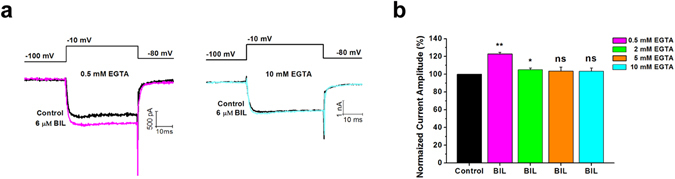
A high concentration of EGTA (2 mM, 5 mM and 10 mM) prevented cells from bilirubin induced enhancement of Ca^2+^ currents. (**a**) Representative traces at −10 mV for currents obtained in the presence or absence of 6 μM bilirubin (black trace) with 0.5 mM EGTA (magenta trace, left panel) and 10 mM EGTA (cyan trace, right panel) in intracellular solutions. Each current was elicited by a 40 ms test pulse to −10 mV from −100 mV. (**b**) The normalized VGCC current amplitude before (black bar) and during application of 6 μM bilirubin with pipette solutions containing 0.5 mM EGTA (magenta bar), 2 mM EGTA (green bar), 5 mM EGTA (orange bar) and 10 mM EGTA (cyan bar). *P < 0.05, **P < 0.01, ns, not significant, one-way ANOVA with Bonferroni post hoc test.

It is well known that residual Ca^2+^ concentration can facilitate subset of VGCCs such as P/Q-type^34, 35^. Our previous work demonstrated that bilirubin can cause a slow rise in intracellular Ca^2+^ concentration via possibly the release of internal Ca^2+^ stores^36^, raising the possibility that bilirubin can enhance P/Q-type Ca^2+^ current by Ca^2+^-dependent facilitation of Ca^2+^ currents. To address this, we added 2 mM, 5 mM and 10 mM EGTA to the pipette solution instead of 0.5 mM EGTA and examined the effect of bilirubin. We found that the current amplitude after bilirubin application (6 μM) was not different from that in the control solution with 5 or 10 mM EGTA (103.6 ± 4.4% of control; p = 0.11, n = 6 or 103.3 ± 3.8% of control; p = 0.39, n = 7). Three of seven cells tested even showed slight inhibition of current amplitude in the presence of bilirubin. Reduction to 2 mM EGTA allowed a small enhancement of current amplitude by bilirubin to emerge (105.0 ± 2.2% of control; p = 0.036, n = 6), but the effect was not as much as 0.5 mM EGTA (Fig. 6a and b). The amplitude of the current density showed no difference with 0.5 and 2 mM EGTA in the absence of bilirubin (37.7 ± 1.4 pA/pF with 0.5 mM EGTA, n = 41; 37.7 ± 1.3 pA/pF with 2 mM EGTA, n = 6; p = 1). The fact that bilirubin induced increase in VGCC current amplitude diminishes with intracellular EGTA as low as 2 mM indicated that bilirubin most likely exerts its enhancing effect on VGCCs via elevating residual Ca^2+^ concentration.

### Calmodulin mediates bilirubin-induced enhancement of VGCC currents

**Figure 7:**
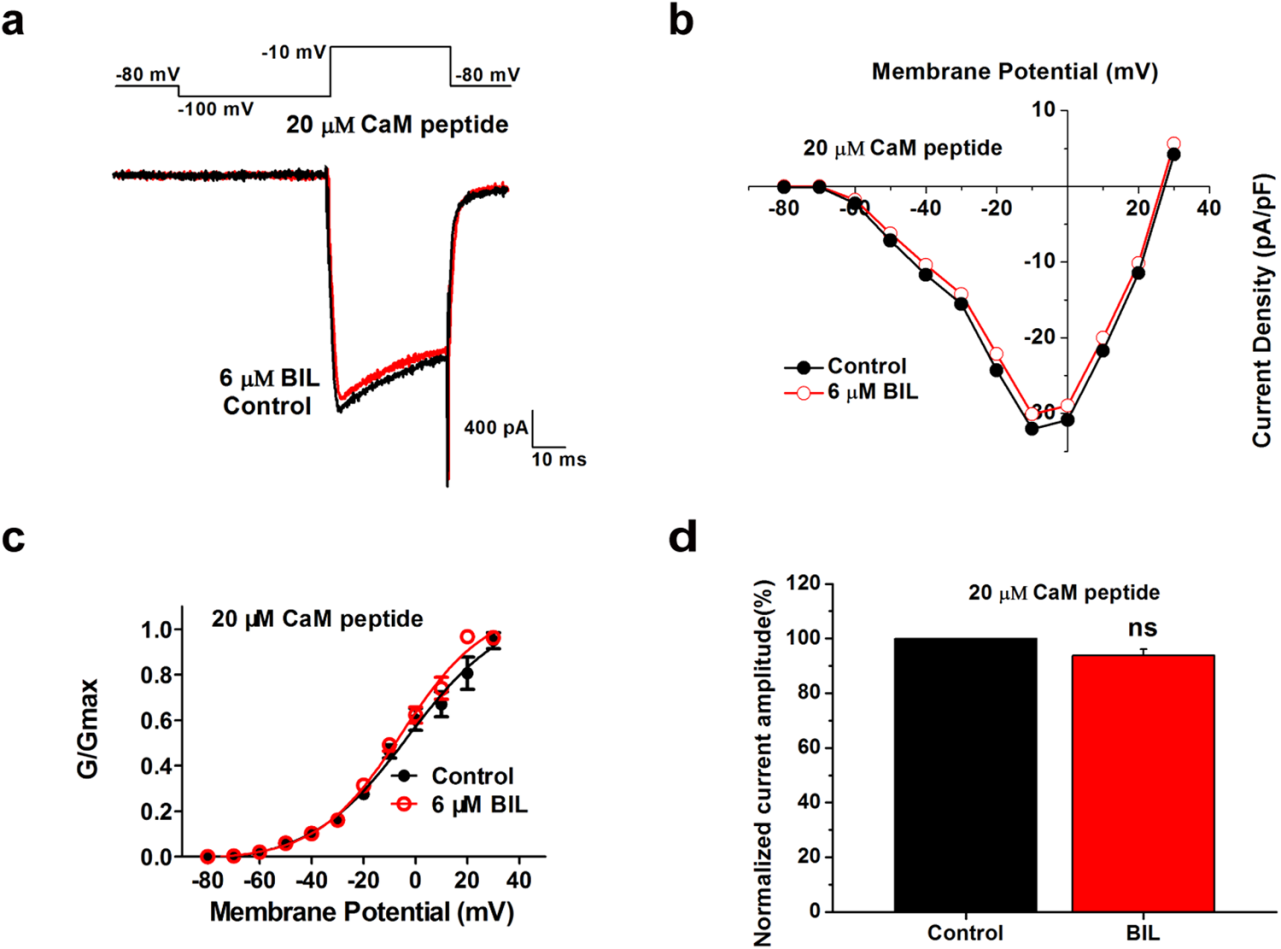
Calmodulin inhibitory peptide affects the amplitude of calcium currents and steady-state activation induced by bilirubin (**a**) Representative traces in control condition (black trace) and bath application of 6 μM bilirubin (red trace) with 20 μm CaM inhibitory peptide. Each current was elicited by a 40 ms test pulse to −10 mV from −100 mV. (**b**) Current-voltage relationships from data in A measured at the peak current density showed VGCCs currents in control solution (black trace) and 6 µM bilirubin (red trace). (**c**) Mean conductance-voltage relationships for peak calcium currents from data in A were obtained by a 1 s prepulse to various potentials (10 mV increments) followed by a 50 ms test pulse to −10 mV are shown. Steady-state activation of VGCCs in control (black circles) and bilirubin group (red circles) were fit to a Boltzmann relationship. Exposure of bushy neurons to 6 μM bilirubin caused no significantly effect on the steady-state activation curve. (**d**) The normalized VGCC current amplitude before (black bar) and during application of 6 μM bilirubin (red bar) from nine neurons. There was a smaller current in amplitude with application of bilirubin with CaM inhibitory peptide. ns, not significant, Student’s paired t test.

Ca^2+^-dependent facilitation of VGCC currents is mediated by calmodulin binding to IQ motif of the C-terminal of α subunit, e.g. α1 A subunit, the main pore-forming subunit of the P/Q-type VGCCs^37^. To determine if such a mechanism was involved in bilirubin induced enhancement of P/Q-type VGCC currents, we made use of a specific calmodulin inhibitory peptide (CaM peptide), which is a 17-residue peptide based on the calmodulin-binding domain of myosin light chain kinase, binding to calmodulin with high affinity (Kd = 6 pM). By including 20 μM membrane-impermeable CaM inhibitory peptide directly into the pipette solution, we found that bilirubin induced enhancement of calcium currents was completely blocked (93.9 ± 2.6% of control, p = 0.053, n = 9) (Fig. 7a,b and d) while voltage-dependent activation of VGCCs was unaffected by the presence of CaM inhibitory peptide (Fig. 7c). The mean V_0.5_ values were −2.9 ± 3.9 mV in control and −5.4 ± 1.9 mV in bilirubin (p = 0.876, n = 9) with no change in the slope factor (control, k = 18.7 ± 3.1; bilirubin, k = 16.2 ± 1.6; p = 0.761, n = 9). These results suggested that residual Ca^2+^ facilitates VGCC currents likely via calmodulin-dependent mechanisms.

### Bilirubin boosts Ca^2+^ load through VGCC currents evoked by pseudo-APs

**Figure 8:**
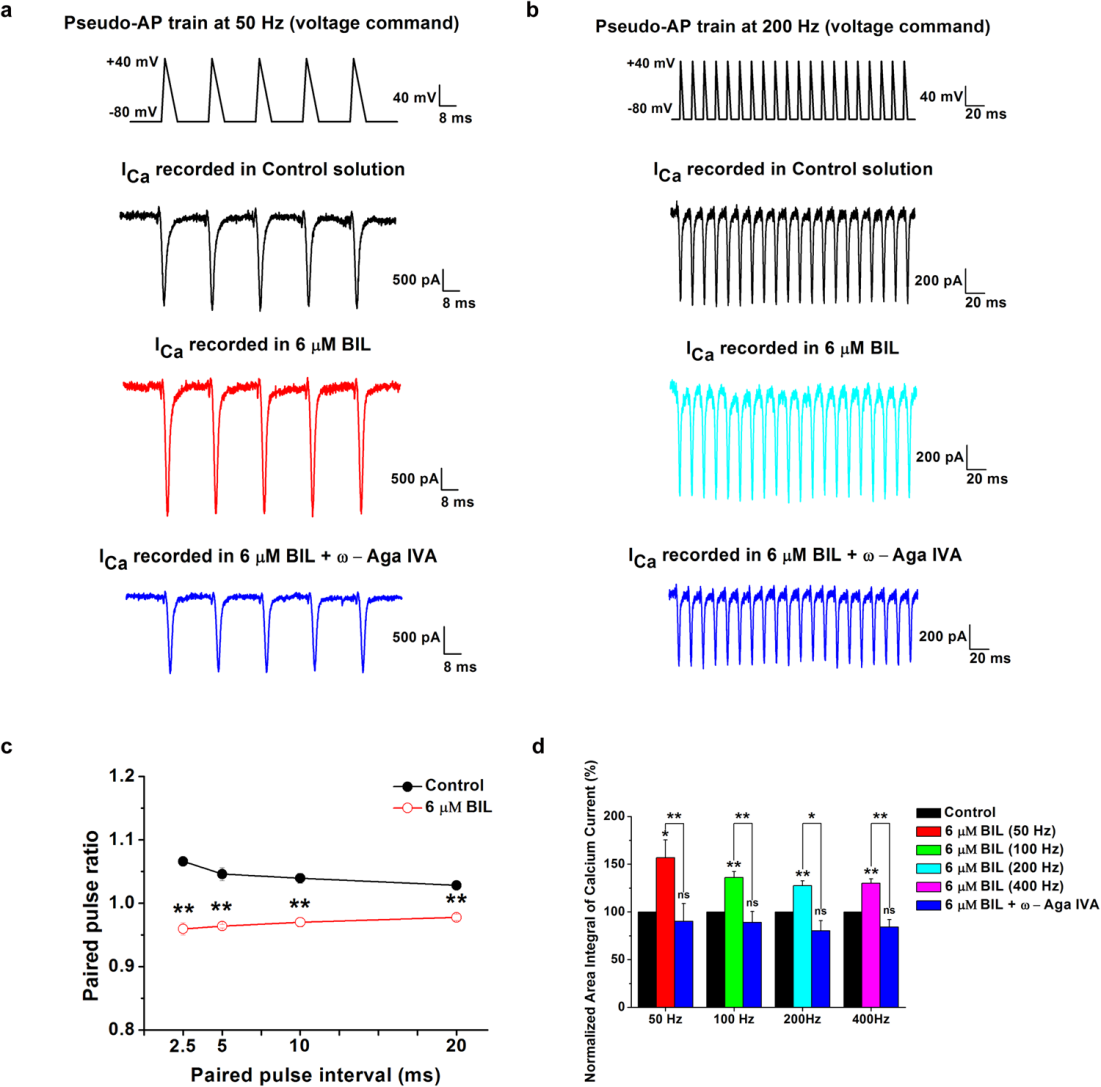
Bilirubin-induced enhancement in Ca^2+^ integrals through P/Q-type VGCC by pseudo AP trains. Example recordings of calcium currents in response to 50 Hz and 200 Hz AP like voltage ramps (AP waveform: −80 to + 40 mV, rise time: 2 ms, decay time: 6 ms; Top traces in **a** and **b**) in the presence of Ca^2+^ buffer EGTA at low concentration (0.5 mM) in pipette solution. The control solution was applied for 5 min, followed by the bath application of 6 μM bilirubin for 6 min and 500 nM ω- Agatoxin IVA for another 5 min. The paired pulse ratio of the amplitude of the second and the first spike at 50 Hz, 100 Hz, 200 Hz and 400 Hz in the absence (**c**, black circle) and presence of 6 μM bilirubin (**c**, red circle) described the difference in the facilitation of VGCCs. The area integral of calcium current evoked by AP trains increased at 50 Hz (**d**, red bar), 100 Hz (**d**, green bar), 200 Hz (**d**, cyan bar) and 400 Hz (**d**, magenta bar), and was all attenuated after applying ω- Agatoxin IVA (**d**, blue bars). *P < 0.05, **P < 0.01, ns, not significant, one-way ANOVA followed by Bonferroni post hoc test.

VGCCs mediate Ca^2+^ influx in response to APs^11^ that propagate and code information in the mammalian brain. For example, bushy cells in AVCN are a main source of excitatory outputs to more central auditory nuclei. These neurons have primary-like responses capable of high-frequency firings in response to oscillatory characteristic of auditory input. To directly investigate whether bilirubin affects Ca^2+^ entry activated by APs, we evoked calcium currents (Ic_a_) by trains of pseudo-AP voltage-clamp templates at different frequencies (50, 100, 200, 400 Hz) with their waveforms mimicking native spikes from these neurons in rats at P8-10. We found that calcium currents activated by AP train template enhanced in all cases after exposure to bilirubin. The area integral of calcium current evoked by AP trains increased from 156.8 ± 18.8% (n = 9) at 50 Hz to 130.1 ± 4.5% (n = 9) at 400 Hz to that of control solutions. After application of 500 nM ω-Aga IVA, we found that bilirubin induced enhancement in the area integral of calcium current was close to control levels at all frequencies from 90.3 ± 18.6% (n = 5) at 50 Hz to 84.2 ± 7.9% (n = 5) at 400 Hz of control solutions (Fig. 8a,b and d). When we examined paired-pulse ratios of VGCC currents evoked by the first two spikes (i.e. P2/P1) for stimuli at intervals of 2.5, 5, 10, and 20 ms, we found typically VGCC currents show pair-pulse facilitation under control condition but instead pair-pule depression after bilirubin application, with P2/P1 ratio being significantly smaller in the presence of bilirubin than those in the absence of bilirubin at any inter-pulse interval (Fig. 8c). Collectively, our results suggested that acute exposure to bilirubin more robustly enhances P/Q-type calcium currents by physiologically relevant spiking trains than that by voltage-steps, likely contributing to Ca^2+^ overload and potentially Ca^2+^-dependent neurotoxicity.

## Discussion

The major finding of this study is that acute bilirubin administration increases the current amplitude of VGCCs in bushy neurons of AVCN in neonatal rats, via selective enhancement of the current mediated by P/Q subtype. This increase is particularly prominent during high-frequency pseudo APs, implicating that the excess Ca^2+^ influx into auditory neurons potentially contributes to hyperbilirubinemia-dependent neurotoxicity in early developing brain.

Bilirubin neurotoxicity is determined by the free fraction of unconjugated bilirubin which can easily cross the blood–brain barrier to exert toxic effects^38, 39^. Cerebrospinal fluid (CSF) bilirubin levels are under 8.55 μmol/L in normal neonates^40^. However, a danger zone of CSF unbound bilirubin (1.71 to 2.57 μmol/L) was observed in presence of kernicterus of newborn infants^41^ and *in vitro* studies suggest a free bilirubin concentration in the range of 71–770 nmol/L can induce neurotoxicity^42^. In this study, application of 3 μM and 6 μM bilirubin in brain slices could significantly augment VGCC currents, suggesting such concentrations of bilirubin likely promote Ca^2+^ overload in neonates with clinical hyperbilirubinemia.

Bilirubin toxicity has been shown to alter intracellular Ca^2+^ regulation^43^. It is well accepted that the influx of extracellular Ca^2+^ into cells is accomplished via ligand- or voltage-gated calcium-permeable ion channels such as glutamate receptors or VGCCs^44^. In the present study, we used glutamate receptor antagonists to block the Ca^2+^ entry into Ca^2+^-permeable NMDA and AMPA receptors due to bilirubin-dependent enhancement of glutamate release as shown in our previous work^7^. Thus, the enhancement of calcium currents induced by bilirubin was unlikely caused by Ca^2+^-dependent facilitation of VGCCs due to Ca^2+^ influx via glutamate receptors. We have previously showed that bilirubin can trigger Ca^2+^ release from internal stores^36^, which may in turn elevate intracellular Ca^2+^ level and facilitate calcium currents. Increased intracellular Ca^2+^ may bind to a major regulatory protein, calmodulin, acting as an intrinsic calcium sensor that endows the channels with calcium-dependent facilitation^11^. We found that blocking calmodulin with an inhibitory peptide occluded bilirubin-induced enhancement of VGCC currents, indicating an essential role of calmodulin in mediating bilirubin’s effects on VGCCs. However, we cannot exclude the possibility that bilirubin can directly bind to and positively modulate VGCCs as evidenced by a left-shift in their activation curve, or indirectly other targets to increase intracellular Ca^2+^ level and channel activity.

Given the vulnerability of neonatal neurons to bilirubin, it is important to examine whether and how different subtypes of VGCCs are developmentally regulated and respond to acute bilirubin applications. Different from previous study showing no marked changes in the relative contribution of somatic calcium current subtypes to the total current in the same cells over the age range of P6-P14 (in Lister Hooded rats)^27^, we found that the relative proportion of different subtypes of VGCC changes with development in Sprague Dawley rats. T-type VGCCs decreases significantly with age, consistent with the observation that in early stage, neurons have only LVA somatic calcium channels. Later in conjunction with neurite sprouting, neurons express less LVA channels, with eventually predominating with HVA calcium channels^45–48^. In contrast, R-, L-type and N-type VGCCs tend to increase, becoming dominant during development. Surprisingly, we found that P/Q calcium currents contribute a significantly smaller proportion of the total calcium current in bushy neurons and yet exhibited highest sensitivity to bilirubin. Previous study suggests that somatic expression of HVA channels in VCN may be relatively low and stable due to the fact these channels are targeted to presynaptic terminals as a result of their transport away from soma to axons. Aside from differences in rat strains, an extended age span (P4–17) in our study may underpin the difference in developmental changes with respect to the relative contribution of each subtype to total somatic Ca^2+^ currents from that reported in previous study^27^.

We present evidence that acute bilirubin exposure specifically enhances currents and channel activities mediated by P/Q-type VGCCs, and this enhancement is most robust during 50 Hz pseudo APs. Given that auditory nerves spike spontaneously *in vivo* before the onset of hearing at similar frequencies to repetitive firings of bushy cells in VCN^49^, we suggest that this enhancement can induce Ca^2+^ overload and contribute to the neurotoxicity associated with hyperbilirubinemia. A collapse of homeostatic Ca^2+^ control in neurons can cause cell death. For instance, apoptosis, the best known form of active cell demise, can be brought about by a loss of Ca^2+^ homeostatic control^50^. It is possible that bilirubin-induced dysregulation of Ca^2+^ triggers distinct parts of the cell death programs, which can then function alone or combine with other signaling pathways to kill the cell, for instance, activating caspases, the mainstream Ca^2+^-dependent apoptosis executioners.

Our data supports the idea that unconjugated bilirubin may argument the Ca^2+^ influx via VGCCs to potentially perturb intracellular Ca^2+^ homeostasis, resulting in activation of downstream targets that ultimately mediate neuronal injury^10^. The particular vulnerability of developmental brain to bilirubin-induced hyperexcitation may be attributed to the low expression levels of endogenous Ca^2+^ buffers in auditory neurons to buffer enhanced Ca^2+^ influx^51^. It has been reported that the endogenous buffer capacity of central neurons is increased during maturation and increase in its buffer capacity can rescue the cognitive deficits^52^. Our observation that high concentration of EGTA in cytoplasm can prevent enhancement of calcium currents from bilirubin is consistent with the observation that Ca^2+^ chelators may protect neurons from early neurodegeneration triggered by excess intracellular Ca^2+^ *in vivo* ^53, 54^. Residual Ca^2+^ would prolong Ca^2+^ channel opening, and enhance facilitation^11^. Knowing Ca^2+^-dependent facilitation is unique to Ca^2+^ entry through P/Q-type VGCCs in nerve terminals^35^, we infer that bilirubin-induced increase in P/Q-type channel currents and robust facilitation particularly during high-frequency spiking, generating much larger Ca^2+^ overload than endogenous buffers can absorb and consequentially neurotoxicity. In contrast, mature neurons express high levels of endogenous calcium chelators in cytoplasm^51, 55, 56^, which may preclude them from neuronal damage as seen in in young neurons.

It is interesting to note that VGCC currents evoked by pseudo AP trains revealed bilirubin exposure expanded the total Ca^2+^ integral in all frequencies tested in this study. It is well known that APs play a crucial role in evoking calcium currents through VGCCs and in transmitter release. It has been confirmed that an AP can effectively open a majority of presynaptic VGCCs (50~90%) in cortical synapses^57, 58^ and immature auditory synapses^59–61^. Knowing P/Q-type VGCCs are particularly important for mediating neurotransmitter release^62, 63^, one can envisage that robust increases of the integral of calcium current or intracellular Ca^2+^ concentration by bilirubin dramatically boost transmitter release from nerve terminals during high-frequency firing^64^, and potentially contribute to the bilirubin-dependent enhancement of glutamate release and hyperexcitability^7^, as well as degeneration of the calyx of Held terminal originated from VCN bushy cells^65^.

In conclusion, our experimental evidence demonstrated that bilirubin enhances the amplitude and integral of calcium currents, which likely contribute to Ca^2+^ overload. Our findings that P/Q-type channel blocker ω-Aga IVA largely attenuated and bilirubin-induced enhancement of Ca^2+^ raise the possibility that P/Q-type channel blocker can act as a promising alternative to treat neonate hyperbilirubinemia. Further work is needed to investigate roles of VGCCs in chronic bilirubin exposure, as VGCCs are of pivotal importance for intracellular homeostasis and neurotransmission at the key central auditory neurons and other fast spiking central neurons vulnerable to bilirubin-induced neuronal injury in newborns.

## Methods

### Ethical Approval

All experiments were performed in accordance with the guiding principles for the care and use of animals, and protocols used in this study were pre-approved by the Animal Ethics Committee of Shanghai Sixth People’s Hospital.

### Slice preparation

Experiments were conducted using brain slices of VCN obtained from postnatal day 4 (P4) to P17 Sprague Dawley rats which were killed by decapitation under sodium pentobarbital (55 mg kg^−1^, i.p.) anesthesia. The brain was then quickly immersed in oxygenated and ice-cold low-calcium, high-magnesium artificial cerebrospinal fluid (aCSF) containing (in mM): 124 NaCl, 5 KCl, 1.2 KH_2_PO_4_, 0.5 CaCl_2_, 3 MgSO_4_, 24 NaHCO_3_, and 10 glucose saturated with 95% O_2_ and 5% CO_2_ at a pH of 7.3 (osmolality of 300–310 mOsm). The brain region containing the VCN was sectioned into transverse slices (300-μm-thick) using a vibratome (VT-1000s, Leica, Germany). Brain slices were pre-incubated in normal aCSF with 95% O_2_ and 5% CO_2_ for 40–60 min at 35–37 °C. Normal aCSF was the same as slicing saline but with 1.3 mM MgCl_2_ and 2.4 mM CaCl_2_. AVCN in slices were visually identified with a 40x water immersion objective attached to an upright microscope (ECLIPSE FN1, Nikon, Japan).

### Electrophysiology

All recordings were made using the whole-cell patch-clamp technique via a patch-clamp amplifier (EPC-10; HEKA, Germany). Bushy cells were visually identified by differential interference contrast (DIC) based on their large, round soma with small dendritic trees in AVCN and fast capacitance transients with typical single-exponential decay. The patch electrodes were fabricated from filamented, thin-wall, 1.5 mm outer diameter, borosilicate capillary glass (World Precision Instruments, Sarasota, FL) and pulled with a vertical pipette puller (P-9; Narishige, Tokyo, Japan). Recording pipettes had resistances of 2–4 MΩ. In voltage-clamp experiments, before compensation of whole-cell capacitance, cells were held at −70 mV and a depolarizing step to +20 mV was applied. Cell capacitance (Cm) was calculated using the equation (1)

1 $${\rm{Cm}}={\rm{Q}}/{\rm{V}},$$

where Q is the charge measured by integrating the capacitive current evoked with the pulse^66^. Series resistance varied from 5 to 10 MΩ. All reported results were from recordings in which approximately 90% of the series resistance could be compensated. Cells showing higher resistances (>10 MΩ) were omitted from the analysis. Calcium current was evoked by various voltage-command paradigms, indicated in the text, and subtraction of capacitive current and leak current was done with the on-line P/4 protocol. Details for pseudo-APs as voltage-clamp paradigms are given in the text and figure legends. For recording VGCC currents, the extracellular solution with Barium (Ba^2+^) as charge carrier instead of Ca^2+^ contained (in mM): 135 TEA-Cl, 10 CsCl, 10 4-AP, 5 BaCl_2_, 10 HEPES, and 10 glucose, and TTX (1 µM), DNQX (40 µM), D-AP-5 (50 µM), bicuculline (10 µM) and strychnine (1 µM) were added to the external solution to block voltage-sensitive sodium currents and glutamatergic and glycinergic synaptic currents. For recording calcium currents with Ca^2+^ as charge carrier, 2 mM CaCl_2_ was added to the extracellular solution instead of 5 mM BaCl_2_. The pipettes were filled with an intracellular solution containing (in mM): 105 CsCl, 40 HEPES, 5 D-glucose, 0.5 EGTA, 2.5 MgCl_2_, 2 MgATP and 0.5 GTP, pH 7.4 with CsOH, except for the experiments in Fig. 6 where EGTA of higher concentrations were used. Current-voltage relationships were obtained by holding cells at −80 mV and hyperpolarizing to −100 mV before stepping to various test potentials from −100 to + 40 mV. Percentage block of these currents was defined as the equation (2)

2 $$[1\,-({\rm{Itest}})/({\rm{Icontrol}})]\,\times \,100,$$

and values from depolarizing steps to −10 mV were used to evaluate calcium current percentage changes. The current density was determined by dividing total current by cell membrane capacitance (pA/pF). The effectiveness of bilirubin on VGCC currents was determined by a series of experiments in which different does of bilirubin (1–6 µM) were applied in the same (or different) bushy neurons. All data were controlled by PatchMaster software (HEKA; 2.9 kHz Bessel filter, sampled at 10 kHz). All experiments were performed at room temperature (23–27 °C).

### Data and Statistical analysis

All data are stored on a personal computer for further off-line analysis. VGCC currents were measured by either current size (pA) or current density (pA/pF), except calcium current over an AP train was calculated by the area integral. In activation and steady-state inactivation curves, peak inward currents obtained from activation protocols and currents recorded from test pulse in inactivation protocols were converted to conductance values using the equation (3)

3 $${\rm{G}}={\rm{I}}/({\rm{Vm}}-{\rm{ECa}}),$$

where *G* is the conductance, *I* is the peak inward current, *Vm* is the membrane potential step used to elicit the response, and *E*_*Ca*_ is the reversal potential for calcium (determined for each cell using the x-axis intercept of a linear fit of the peak inward current responses to the last five voltage steps from 0 mV to + 40 mV of the activation protocol). Activation and steady-state inactivation of VGCCs were then fit by a Boltzmann equation (4)

4 $${\rm{G}}/{\rm{Gmax}}\,=\,1/(1\,+\exp [({\rm{V0}}{\rm{.5}}-{\rm{Vm}})/{\rm{k}}]),$$

where *G* and *Gmax* represent relative conductance as a function of the prepulse potential and *G/Gmax* is the ratio of conductance to maximum conductance, *Vm* represents the inactivating prepulse membrane potential, *V*_*0.5*_ is the potential of half-maximal activation or inactivation, and *k* is the slope factors for the component representing the steepness of the Boltzmann fits. Clampfit 10.2 software (Molecular Devices), Origin 8 (Microcal Software), SigmaPlot12 (Systat Software) and GraphPad Prism 5 (GraphPad Software) were used for data analysis and graphic representation. Statistical analyses were performed using SPSS 17.0 software (SPSS Inc.). For correlation analysis, least-squares linear regression was performed using Origin 8. Experimental data in the text and figures were expressed as means ± SEMs. Raw data were compared for statistical significance using Student’s paired t test, one-way ANOVA followed by Bonferroni post hoc test or two-way ANOVA followed by Bonferroni post hoc tests with p < 0.05 taken as the level of significance.

## Materials

The materials and drugs used in this study included free bilirubin (BIL), 6,7-dinitroquinoxaline-2,3-dione (DNQX), D-2-Amino-5-phosphonopentanoic acid (D-AP-5), bicuculline, strychnine, tetrodotoxin (TTX), VGCC antagonists and calmodulin inhibitory peptide. Chemicals and drugs were purchased from Sigma (St Louis, MO), except where indicated otherwise. Bilirubin was dissolved in 0.1 M NaOH at 1 mM as a stock solution and stored in disposable aliquots for <48 h. TTX and VGCC blockers ω-Aga IVA and ω-CTx GVIA, were purchased from Alomone Labs (Jerusalem, Israel). TTX was prepared in distilled water at 5 mM, and ω-Aga IVA and ω-CTx GVIA were dissolved in water at concentrations of 0.125 mM and 0.25 mM, respectively. Nifedipine and Mibefradil stock solutions were prepared in dimethyl sulfoxide (DMSO) at 5 mM and 0.5 mM, respectively; NiCl_2_ was dissolved in water at 25 mM. Calmodulin inhibitory peptide were purchased from Calbiochem, prepared in distilled water at 4 mM. Bilirubin and nifedipine were kept away from light both in stock solution and among all experiments. Aliquots of stock solutions were kept at −20 °C and were later diluted with final concentration before use. The ultimate concentration of DMSO was <1:5000, a concentration that was found to have no effect on calcium current^67^. Application of drugs was achieved by switching a multi-valve, single-output gravity perfusion system to the experimental chamber at a speed of 1–2 ml min^−1^.
